# CXCL12 and CXCL13 as potential biomarkers for disease severity and recurrence in respiratory syncytial virus bronchiolitis

**DOI:** 10.1038/s41598-025-31062-6

**Published:** 2025-12-08

**Authors:** Lin Zhang, Yuanyu Lv, Zhiao Du, Jiawei Chen, Peng Mo, Xuena Xu, Huiming Sun, Yongdong Yan, Canhong Zhu, Li Huang, Chuangli Hao, Xiuxia Zhou, Heting Dong, Zhengrong Chen

**Affiliations:** 1 Department of Respiratory Medicine, Children’s Hospital of Soochow University, Suzhou, 215003 China; 2 Institute of Pediatric Clinical Research, Children’s Hospital of Soochow University, Suzhou, 215003 China; 3 Pediatric Internal Medicine, Jinan Maternity Child Careand Hospital, Jinan, 250000 China

**Keywords:** Respiratory syncytial virus, Bronchiolitis, Chemokines, CXCL12, CXCL13, Microbiology, Biomarkers

## Abstract

To examine chemokine expression in children with Respiratory Syncytial Virus (RSV) bronchiolitis and evaluate its clinical utility for early warning and prognosis. Five hospitalised RSV bronchiolitis children and five matched controls were studied. To validate findings, 50 RSV infants and 30 controls were assessed for recurrent wheezing after 1 year. Blood leukocyte RNA-seq identified RSV-associated hub genes via GO/KEGG analysis, with flow cytometry confirming chemokine expression. Twelve hub genes were identified, with 712 differentially expressed genes (292 upregulated, 420 downregulated). RSV patients showed elevated CXCL2, CXCL12, CXCL13, CCL13, and CCL24 (*P* < 0.05). CXCL12 was higher in moderate-to-severe cases (Area Under the Curve, AUC = 0.835, 95% CI 0.714–0.956, *P* < 0.05), while CXCL13 was elevated in recurrent wheezers (AUC = 0.851, 95% CI 0.711–0.991, *P* < 0.05). CXCL12 predicted severity, and CXCL13 predicted recurrence (ROC-confirmed, *P* < 0.05). CXCL12 and CXCL13 may serve as biomarkers for assessing RSV bronchiolitis severity and predicting recurrence, aiding early clinical evaluation and prognosis.

## Introduction

Despite fundamental progress in research since the discovery of respiratory syncytial virus (RSV) more than six decades ago, repeated failures in developing effective vaccines and therapeutic strategies to control RSV infection have made it one of the leading causes of infant morbidity and mortality worldwide. During the coronavirus disease 2019 (COVID-19) pandemic, RSV infections declined to low levels due to non-pharmaceutical interventions, but in the post-pandemic era, RSV has resurged with altered clinical characteristics^1–3^. Prevention of RSV infection has become a key focus in pediatric medicine following the COVID-19 pandemic. Current guidelines recommend immunoprophylaxis strategies for RSV bronchiolitis, specifically advocating nirsevimab administration to neonates and infants born during or entering their first RSV season. This prophylaxis aims to pTabrevent RSV-associated acute lower respiratory tract infections (ALRTIs), including bronchiolitis^4^. The pre-F protein vaccine has been approved for adults aged ≥ 65 years, while bivalent pre-F protein vaccines (targeting both RSV-A and B subtypes) for pregnant women and pediatric pre-F vaccines are forthcoming^5^. These advancements are expected to significantly improve RSV prevention and control. This has resulted in an increasing financial burden on families and healthcare systems globally^6^. Furthermore, high-quality epidemiological evidence indicates that early lower respiratory tract infections caused by RSV contribute to subsequent recurrent wheezing and the development of childhood asthma^7,8^. Although RSV is an important respiratory pathogen, the mechanisms linking primary RSV infection to subsequent wheezing and asthma remain poorly understood.

While some progress has been made in identifying reliable biomarkers for RSV bronchiolitis, significant gaps remain in objectively defining disease severity and predicting clinical outcomes. A universal biomarker capable of accurately and consistently predicting disease severity and post-acute outcomes has not yet been discovered. Therefore, the aim of this study is to identify effective biomarkers for RSV infection. Further, we aim to investigate key genes (hub genes) and their signaling pathways associated with RSV bronchiolitis, which will provide a foundation for treatment and subsequent interventions. This will enable pediatricians to predict the clinical course of the disease in a cost-effective manner and monitor the efficacy of new therapeutic strategies.

## Methods

### Study design and participant characteristics

This study initially enrolled five hospitalised children with RSV bronchiolitis and five age- and sex-matched controls (elective surgery) from Soochow University Children’s Hospital between March 2021 and December 2021 for preliminary RSV high-throughput sequencing, followed by the inclusion of 50 cases of RSV bronchiolitis and 30 matched controls. The specific inclusion criteria are as follows: the case group comprised children aged from 1 month to 2-years with RSV-positive nasopharyngeal aspirates who met the bronchiolitis clinical criteria, while controls were age- and sex-matched children who underwent elective surgeries (e.g., hernia and phimosis) without any history of recent infections or wheezing. Exclusion criteria included prematurity, major organ dysfunction, congenital/respiratory diseases, systemic disorders, malignancies, genetic conditions, recent antibiotic/steroid use, or co-infections. Based on the established clinical guidelines, Based on established clinical guidelines, a scoring tool that integrates the Wang score and the modified Tal score has been developed to assess the severity of bronchiolitis for clinical practice. The specific scoring criteria are shown in Table 1^9–11^, the 50 enrolled patients were classified into the mild (n = 24) and moderate-to-severe (n = 26) groups according to disease severity, which was determined by evaluating the following six clinical parameters: feeding volume, respiratory rate, chest wall retractions, nasal flaring or grunting, oxygen saturation, and mental status, with the presence of any one or more of these criteria indicating moderate-to-severe bronchiolitis. All moderate-to-severe bronchiolitis cases were managed within standard pediatric respiratory care without intensive care unit (ICU) transfer. “Recurrence” refers to repeated episodes of wheezing following the initial RSV bronchiolitis.Recurrent wheezing was defined as ≥ 2 wheezing episodes requiring medical attention within 1 year of the index bronchiolitis.Wheezing outcomes were defined as follows: (1) No recurrence: absence of wheezing episodes during the 1-year follow-up; (2) Recurrent wheezing: ≥ 1 wheezing episode post-discharge within 1 year. After enrolment, parents were instructed to closely monitor wheezing (verified by the hospital/community clinic records to minimize recall bias), provide accurate contact information (phone/address), and participate in quarterly follow-ups for 1 year; thus, ensuring 100% retention. Wheezing recurrence was categorised as follows: recurrent (n = 18) and non-recurrent (n = 32) within 1 year post-discharge, there were no cases lost to follow-up. Clinical data, including age, gender, disease course, and laboratory results (e.g., blood count and C-reactive protein (CRP)), were collected. Pulmonary function (Time to Peak Tidal Expiratory Flow to Total Expiratory Time ratio (TPTEF/TE) and Volume to Peak Expiratory Flow to Expiratory Volume ratio (VPEF/VE)) was graded as mild (23–27%), moderate (15–22%), or severe (< 15%).

**Table 1:** Bronchiolitis severity scoring scale. After admission, the patient’s severity was assessed based on respiratory rate, presence of audible rales, chest wall retractions, oxygen saturation, and general condition (feeding volume, irritability, lethargy, etc.). The total score was calculated as follows: mild severity, 0–5 points; moderate severity, 6–9 points; and severe severity, 10–15 points. Based on the scoring system, the patients were categorized into groups: 24 cases were assessed as mild, and 26 cases as moderate to severe.

Score	0	1	2	3
Feeding volume	Normal	Decreased to more than 75% of normal, mild irritability	Decreased to 50–75% of normal, obvious irritability	Decreased to less than 50% of normal, extremely irritable, drowsy, comatose
Respiratory rate	< 30 breaths/min	30–45 breaths/min	> 45–60 breaths/min	> 60 breaths/min
Inspiratory retraction of the chest wall	None	Mild intercostal retraction is relatively obvious	Moderate intercostal retraction	Extremely obvious intercostal retraction accompanied by head—nodding breathing or suprasternal retraction
Oxygen saturation	≥ 95%	92–94%	90–91%	≤ 89%
Wheezing and/or rales	None	Only audible during expiration	Audible in both phases of respiration (on auscultation only)	Audible in both phases of respiration (not relying on stethoscope)

### Specimen collection and analysis

Peripheral venous blood (2 ml) was drawn from 50 RSV-positive children and 30 controls within 24 h of admission, prior to medication. Samples were anticoagulated, left to stand for 30 min, and centrifuged at 4 °C and 3000 rpm for 10 min. The upper pale-yellow plasma layer was aspirated using a pipette, aliquoted into 1.5 ml EP tubes, labeled, and matched with corresponding clinical data. Plasma samples were immediately stored at -80 °C after centrifugation (3000 rpm) and were avoided repeated freeze–thaw cycles. If precipitation occurred during storage, samples were re-centrifuged prior to use.

Based on prior RSV RNA-seq results, a customized Human Proinflammatory Chemokine Panel 2 (12-plex) kit from BioLegend (USA) was employed. Plasma levels of CXCL2, CXCL12, CXCL13, CCL1, CCL7, CCL8, CCL13, CCL18, CCL19, CCL22, CCL24, and CX3CL1 were measured using a bead-based immunoassay. Capture beads and phycoerythrin (PE)-conjugated chemokine-specific antibodies were mixed, then incubated with recombinant standards and test samples to form sandwich complexes. Fluorescence intensity of PE was detected via flow cytometry, enabling differentiation of distinct chemokine-specific beads.

*Standard operating procedure*: (1) Preparation of Standard Curve: Reconstitute the standard lyophilicate by transferring the vial of white standard microspheres to a 15 mL polypropylene tube, designated as the “Stock Solution.” Add 2 mL of Assay Diluent and mix thoroughly. Prepare a serial dilution series in flow cytometry tubes pre-labeled with dilution factors (1:2, 1:4, 1:8, 1:16, 1:32, 1:64, 1:128, 1:256). Pipette 300 µL of Assay Diluent into each tube. Transfer 300 µL from the Stock Solution into the 1:2 dilution tube. Mix thoroughly by pipetting. Serially transfer 300 µL from the 1:2 tube to the 1:4 tube, mix, and continue this process sequentially through the 1:256 dilution tube. (2) Preparation of Capture Bead Mixture Vortex: each vial of capture beads for 3–5 s prior to use.In a single flow cytometry tube, designated as the “Mixed Beads,” combine 10 µL of each capture bead for the total number of tests (including standard and sample tubes). Vortex the mixture thoroughly. Centrifuge the Mixed Beads tube at a recommended speed for 5 min. Carefully aspirate the supernatant. Resuspend the bead pellet in an equivalent volume of Enhancement Buffer. Vortex thoroughly and incubate the tube for 30 min at room temperature, protected from light. (3) Sample Dilution: Dilute test samples to an appropriate concentration using Assay Diluent. Mix thoroughly to ensure homogeneity. (4) Assay Procedure (Immunofluorescence Staining): Aliquot 50 µL of the thoroughly mixed Capture Bead suspension into an appropriate number of clean flow cytometry tubes. Add 50 µL of the corresponding chemokine standard dilution to the respective tubes. Add 50 µL of the prepared test sample to the designated sample tubes. Add 50 µL of Phycoerythrin (PE)-conjugated Detection Antibody to all assay tubes. Incubate all tubes for 3 h at room temperature, protected from light. Add 1 mL of Wash Buffer to each tube. Centrifuge at 200 × g for 5 min. Carefully aspirate the supernatant from each tube. Resuspend the bead pellet in 300 µL of Wash Buffer. (5) Data Acquisition: Vortex each tube thoroughly for approximately 3–5 s to resuspend the beads. Acquire samples immediately on a flow cytometer. (6) Data Analysis: Analyze acquired sample data using the Bio Legend LEGEND plex data analysis software. The software will automatically generate a standard curve and calculate the precise concentration of each chemokine in the test samples.

Written informed consent was obtained from the legal guardians of the subjects. All procedures were conducted in accordance with the principles of the Declaration of Helsinki and approved by the Ethics Committee of Children’s Hospital of Soochow University (No. 2022CS108).

### Statistical analysis

All data were analyzed using SPSS 26.0 and GraphPad Prism 8.0. Categorical data were presented as n (%) and compared using Chi-square or Fisher’s exact test. Continuous data were tested for normality; normally distributed data were expressed as mean ± SD and compared using t-test or ANOVA, while non-normal data were presented as median (Interquartile Range, IQR) and analyzed using Mann–Whitney U or Kruskal–Wallis H test. Correlations among chemokines (CXCL2, CXCL12, CXCL13, CCL13, CCL24) and their associations with clinical parameters were assessed using Pearson correlation. Binary logistic regression was performed with these chemokines as independent variables and disease severity/recurrent wheezing as dependent variables. ROC curve analysis was used to evaluate the diagnostic value of CXCL12 and CXCL13, with optimal cut-off values determined by maximum Youden index. For multiple comparisons, the significance level was adjusted using the Bonferroni correction method. Statistical significance was set at *P* < 0.05.

## Results

### Differential gene screening findings

**Fig. 1:**
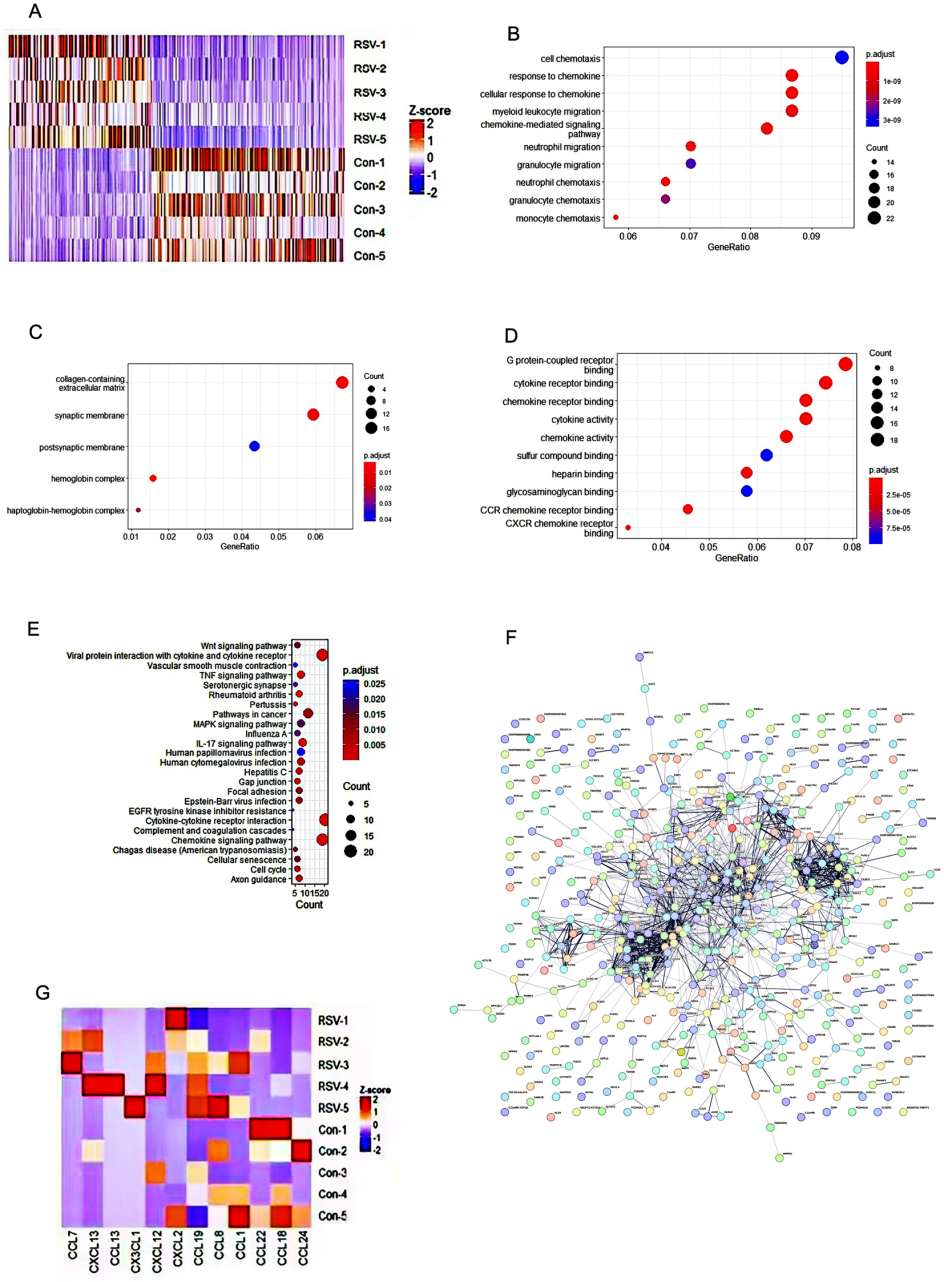
The screening process of hub genes related to RSV infection. (**A**) Clustering heatmap of the bioinformatics analysis results of RNA sequencing for five patient samples versus five control samples. (**B**–**D**) Differentially expressed genes (DEGs) in gene ontology (GO). (**E**) Differentially expressed genes (DEGs) in KEGG. (**F**) Protein–protein interaction (PPI) network of differentially expressed genes (DEGs). (**G**) Visualization heatmap of the association between key gene modules and phenotypes.

Differential gene screening was performed to identify differentially expressed genes (DEGs). The threshold for DEGs was set as false discovery rate (FDR) ≤ 0.05 and (fold change) |FC|≥ 2. The R package DESeq2 was used for differential expression analysis between the case and control groups to identify reliable DEGs. A total of 712 DEGs were identified, including 292 upregulated genes and 420 downregulated genes (Fig. 1A).

### Results of gene ontology (GO) and Kyoto encyclopedia of genes and genomes (KEGG) enrichment analyses of differential genes

Functional annotation and pathway enrichment analysis were performed on the DEGs. GO enrichment analysis revealed that the DEGs were primarily involved in biological processes such as cellular chemotaxis in response to chemokines, cellular response to chemokines, myeloid leukocyte migration, chemokine-mediated signaling pathways, neutrophil migration, granulocyte migration, neutrophil chemotaxis, and monocyte chemotaxis. Additionally, they were associated with the extracellular matrix containing collagen, synaptic membrane, postsynaptic membrane regulation, G protein-coupled receptor activity, cytokine receptor binding, chemokine receptor binding, cytokine activity, and chemokine activity signalling pathways (Fig. 1B–D). KEGG pathway analysis indicated that the DEGs were mainly involved in pathways such as viral protein interactions with cytokines and cytokine receptors, cytokine-cytokine receptor interactions, cytokine signalling in the immune system, adaptive immune system, and neutrophil degranulation. Protein–protein interaction network analysis identified pathways related to interactions between viral proteins and chemokines or cytokine receptors (Fig. 1E).

### Screening findings of hub genes associated with RSV infection

First, the 712 DEGs were imported into the STRING database to construct a protein–protein interaction (PPI) network, which was visualised and analysed using Cytoscape software (Cytoscape3.7.2, https://string-db.org/). Key PPI modules were identified (Fig. 1F). Genes within these modules were primarily involved in biological immune-related processes such as cell cycle regulation, interactions between viral proteins and cytokines or their receptors, and kinase cascade reactions. Similarly, the R package WGCNA was used to analyse highly correlated genes and co-expression networks in RSV. Interactions between gene modules were analysed, and the relative expression heatmap (Eigengene-adjacency-heatmap) of genes from different modules was used to illustrate correlations between gene modules. Core modules with significant differences were identified, and high-risk Hub genes were extracted. Ultimately, the following 12 genes were jointly identified as potential Hub genes: CXCL2, CXCL12, CXCL13, CCL1, CCL7, CCL8, CCL13, CCL18, CCL19, CCL22, CCL24, and CX3CL1 (Fig. 1G).This study focused on highly clustered protein groups, with select functional modules prioritised for subsequent research. In Subfigure 1G, the Visualization Heatmap of the Association between Key Gene Modules and Phenotypes shows that although the number of overexpressed genes (highlighted in red) was relatively low across the five RSV cases—likely due to the small sample size—their expression levels were significantly higher than those in the control group. This observation led to a preliminary hypothesis, prompting further clinical validation with an expanded cohort.

### Demographic distribution and clinical characteristics of children with RSV bronchiolitis

A total of 50 children with RSV bronchiolitis were enrolled for chemokine detection, including 30 males and 20 females, with a median age of 4.8 months (interquartile range [IQR]: 3.6, 8.8). The control group consisted of 30 children, including 19 males and 11 females, with a median age of 8.8 months (IQR: 3.1, 11.8). There were no significant differences in age or gender between the RSV bronchiolitis group and the control group (*P* > 0.05), which indicated comparability. Pulmonary function tests were performed on 11 children in the RSV bronchiolitis group (Table 2).

**Table 2:** Population distribution and clinical characteristics of children with RSV bronchiolitis.

Clinical features	Children with RSV bronchiolitis	Control group	*P*
Age [M (P25, P75), months]	4.8 (3.6,8.8)	8.8 (3.1,11.8)	0.124
Sex, male [n (%)]	30 (60.0)	19 (63.3)	0.767
Family history of asthma or allergic disease [n (%)]	9 (18.0)	–	–
With other allergic diseases [n (%)]	19 (38.0)	–	–
The disease degree		–	–
Mild [n (%)]	24 (48.0)	–	–
Moderate to severe severity [n (%)]	26 (52.0)	–	–
Recurrent wheezing or not		–	–
Recurrent wheeze [n (%)]	18 (36.0)	–	–
No recurrent wheezing [n (%)]	32 (64.0)	–	–
Days of hospitalization [(x ± s), days]	8.2 ± 2.5	–	–

### Comparison of plasma chemokine levels between the RSV bronchiolitis group and healthy control group

**Fig. 2:**
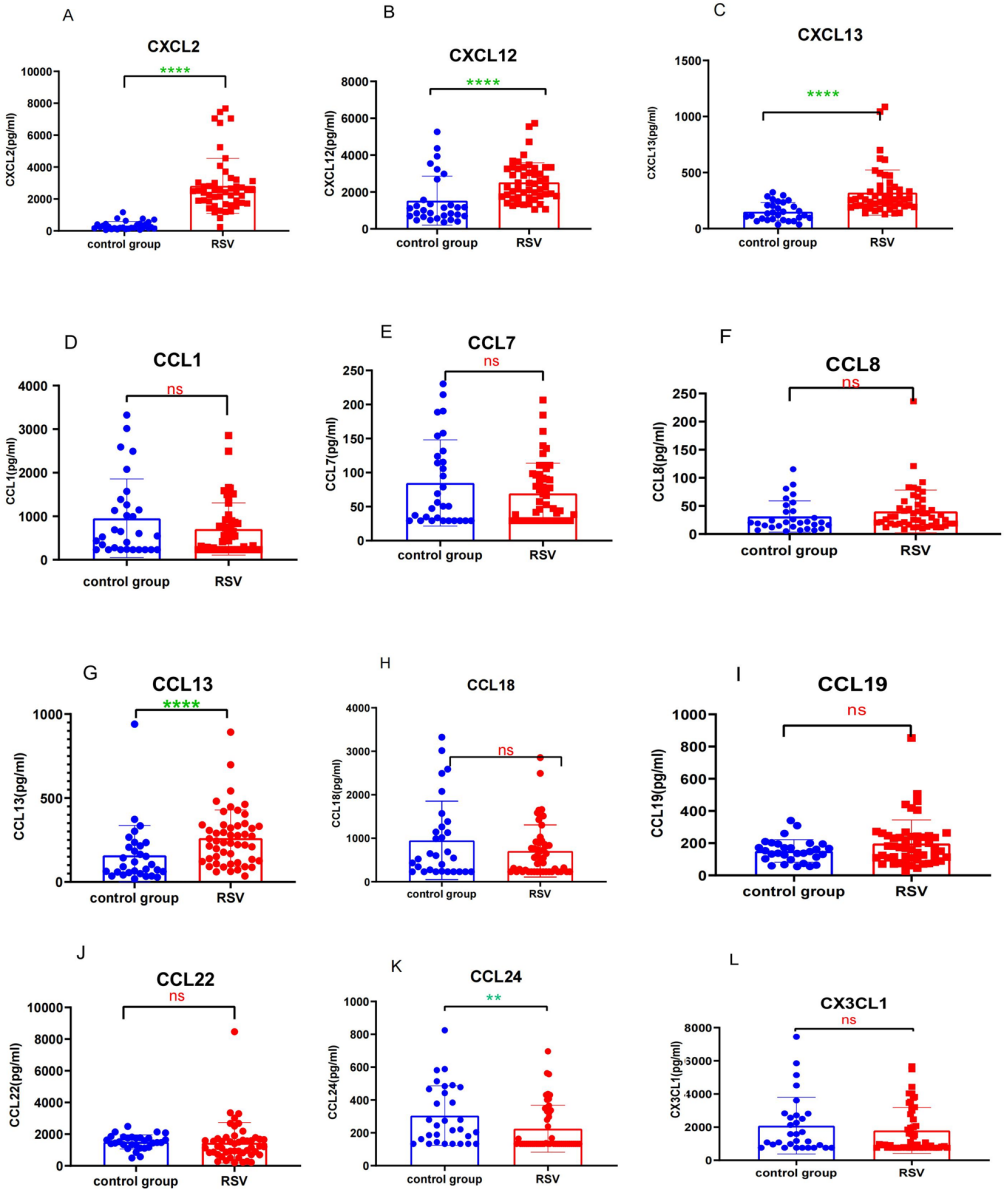
Comparison of plasma pro-inflammatory chemokine levels between the RSV bronchiolitis group and healthy group. e.g. ns (*p* > 0.05); **(*p* < 0.01); ****(*p* < 0.0001.

CXCL2, CXCL12, CXCL13, CCL1, CCL7, CCL8, CCL13, CCL18, CCL19, CCL22, CCL24, and CX3CL1 were detected in the peripheral blood of both the RSV bronchiolitis group and the control group. The plasma levels of CXCL2 in the RSV bronchiolitis group were significantly higher than those in the control group [2460.47 (1745.79, 3055.59) pg/mL vs. 217.12 (160.72, 440.89) pg/mL; *P* < *0.001*]. Similarly, plasma CXCL12 levels were significantly elevated in the RSV bronchiolitis group compared to the control group [2261.05 (1737.67, 3263.10)pg/mL vs.1061.29 (683.40, 1846.43) pg/mL; *P* < *0.001*]. Plasma CXCL13 levels were also significantly higher in the RSV bronchiolitis group [258.76 (199.19, 354.66) pg/mL vs. 127.68 (82.43, 214.50) pg/mL; *P* < *0.001*], as were plasma CCL13 levels [239.33 (125.11, 335.76) vs. 97.69 (54.38, 219.80) pg/mL; *P* < *0.001*]. In contrast, plasma CCL24 levels were significantly lower in the RSV bronchiolitis group compared to the control group [133.02 (121.46, 332.92) pg/mL vs. 232.94 (140.59, 469.12) pg/mL; *P* < *0.01*]. No significant differences were observed in the plasma levels of CCL1, CCL7, CCL8, CCL18, CCL19, CCL22, and CX3CL1 between the two groups (*P* > 0.05) (Fig. 2).

### Comparison of plasma chemokine levels in patients with different severities of RSV bronchiolitis

**Fig. 3:**
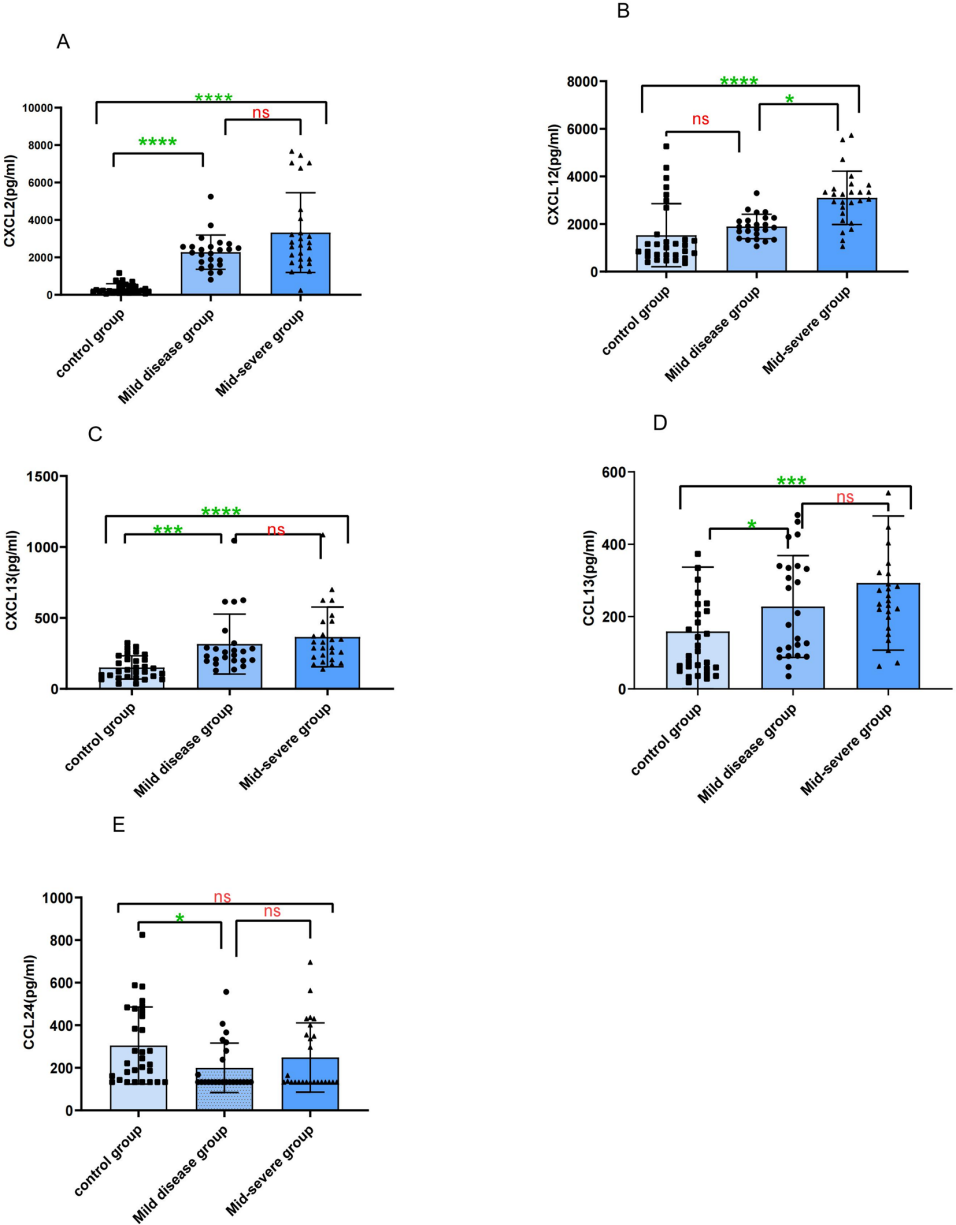
Comparison of plasma chemokine levels between different severities of RSV bronchiolitis groups and healthy control group. e.g. ns (*p* > 0.05); * (*p* < 0.05); ***(*p* < 0.001); ****(*p* < 0.0001).

In this study, 50 children with RSV bronchiolitis were categorised into the mild (24 cases) and moderate-to-severe (26 cases) groups based on disease severity (4). These two groups, along with a healthy control group (30 cases), were compared pairwise with respect to the levels of 12 plasma chemokines. The results showed that plasma CXCL12 levels in the moderate-to-severe RSV bronchiolitis group were significantly higher than those in the healthy control group [3146.48 (2373.99, 3516.18) vs. 1061.29 (683.40, 1846.43) pg/mL; *P* < *0.001*] and the mild group [3146.48 (2373.99, 3516.18) vs. 1865.79 (1440.73, 2232.22) pg/mL; *P* < 0.001]. Plasma levels of CXCL2, CXCL13, and CCL13 were higher in the RSV bronchiolitis group compared to the control group, but no significant differences were observed between the mild and moderate-to-severe groups (*P* > 0.05). Plasma CCL24 levels were higher in the control group compared to the mild RSV bronchiolitis group, with no significant differences between the mild and moderate-to-severe groups (*P* > 0.05). No significant differences were observed in the plasma levels of CCL1, CCL7, CCL8, CCL18, CCL19, CCL22, and CX3CL1 between the RSV bronchiolitis group and the control group, or between the mild and moderate-to-severe groups (*P* > 0.05) (Fig. 3).

### Comparison of plasma chemokine levels between the non-recurrent wheezing and recurrent wheezing groups in children with RSV bronchiolitis

**Fig. 4:**
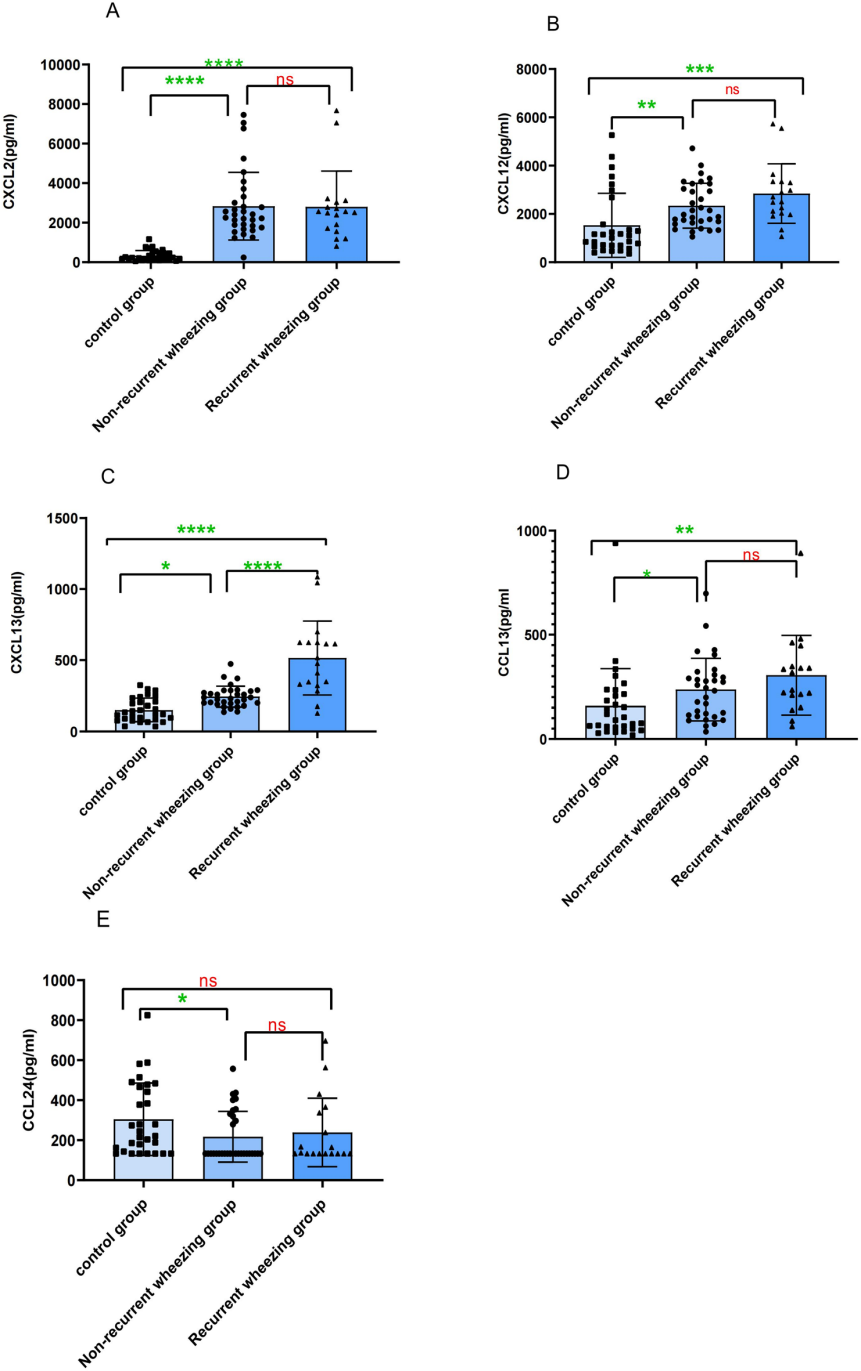
Comparison of plasma pro-inflammatory chemokine levels in plasma between children with RSV bronchiolitis in the non-recurrent wheezing group and recurrent wheezing group. e.g. ns (*p* > 0.05); *(*p* < 0.05); **(*p* < 0.01;) ***(*p* < 0.001); ****(*p* < 0.0001).

Based on the 1-year follow-up, children with RSV bronchiolitis were divided into the non-recurrent wheezing (32 cases) and recurrent wheezing (18 cases) groups. These groups, along with a healthy control group, were compared pairwise with respect to the levels of 12 plasma chemokines. The results showed that plasma CXCL13 levels in the recurrent wheezing group were significantly higher than those in the healthy control group [497.50 (328.37, 624.10) vs. 127.68 (82.43, 214.50) pg/mL; *P* < 0.001] and the non-recurrent wheezing group [497.50 (328.37, 624.10) vs. 234.20 (196.40, 285.90) pg/mL; *P* < 0.001]. Plasma levels of CXCL2, CXCL12, and CCL13 were higher in the RSV bronchiolitis group compared to the control group, but no significant differences were observed between the non-recurrent wheezing and recurrent wheezing groups (*P* > 0.05). Plasma CCL24 levels were higher in the control group compared to the non-recurrent wheezing RSV bronchiolitis group, with no significant differences between the non-recurrent wheezing and recurrent wheezing groups (*P* > 0.05). No significant differences were observed in the plasma levels of CCL1, CCL7, CCL8, CCL18, CCL19, CCL22, and CX3CL1 between the RSV bronchiolitis group and the control group, or between the non-recurrent wheezing and recurrent wheezing groups (*P* > 0.05) (Fig. 4).

### Correlation of plasma CXCL2, CXCL12, CXCL13, CCL13, and CCL24 levels with disease severity and recurrent wheezing

In children with RSV bronchiolitis, plasma CXCL12 levels showed a positive correlation with disease severity (*r* = 0.580; *P* < 0.001), indicating that higher plasma CXCL12 levels were associated with more severe RSV bronchiolitis. In contrast, no significant correlations were observed between the plasma levels of CXCL2, CXCL13, CCL13, or CCL24 and disease severity (*P* > 0.05) (Table 3). Plasma CXCL13 levels were positively correlated with recurrent wheezing (*r* = 0.598; *P* < 0.001). However, no significant correlations were found between the plasma levels of CXCL2, CXCL12, CCL13, or CCL24 and recurrent wheezing (*P* > 0.05) (Table 4).

**Table 3:** Correlation of plasma CXCL2, CXCL12, CXCL13, CCL13, CCL24 and severity of disease. ***P < 0.001.

Index	r	*P*
CXCL2	0.206	0.071
CXCL12	0.580	< 0.001***
CXCL13	0.121	0.403
CCL13	0.196	0.172
CCL24	0.172	0.233

**Table 4:** Correlation of plasma CXCL 2, CXCL12, CXCL13, CCL 13, and CCL 24 and recurrent wheezing. ***P < 0.001.

Index	r	*P*
CXCL2	0.014	0.921
CXCL12	0.176	0.221
CXCL13	0.598	< 0.001***
CCL13	0.188	0.192
CCL24	0.147	0.309

### Correlation of plasma CCL13, CCL24, CXCL13, CXCL12, and CXCL2 levels with clinical indicators

**Fig. 5:**
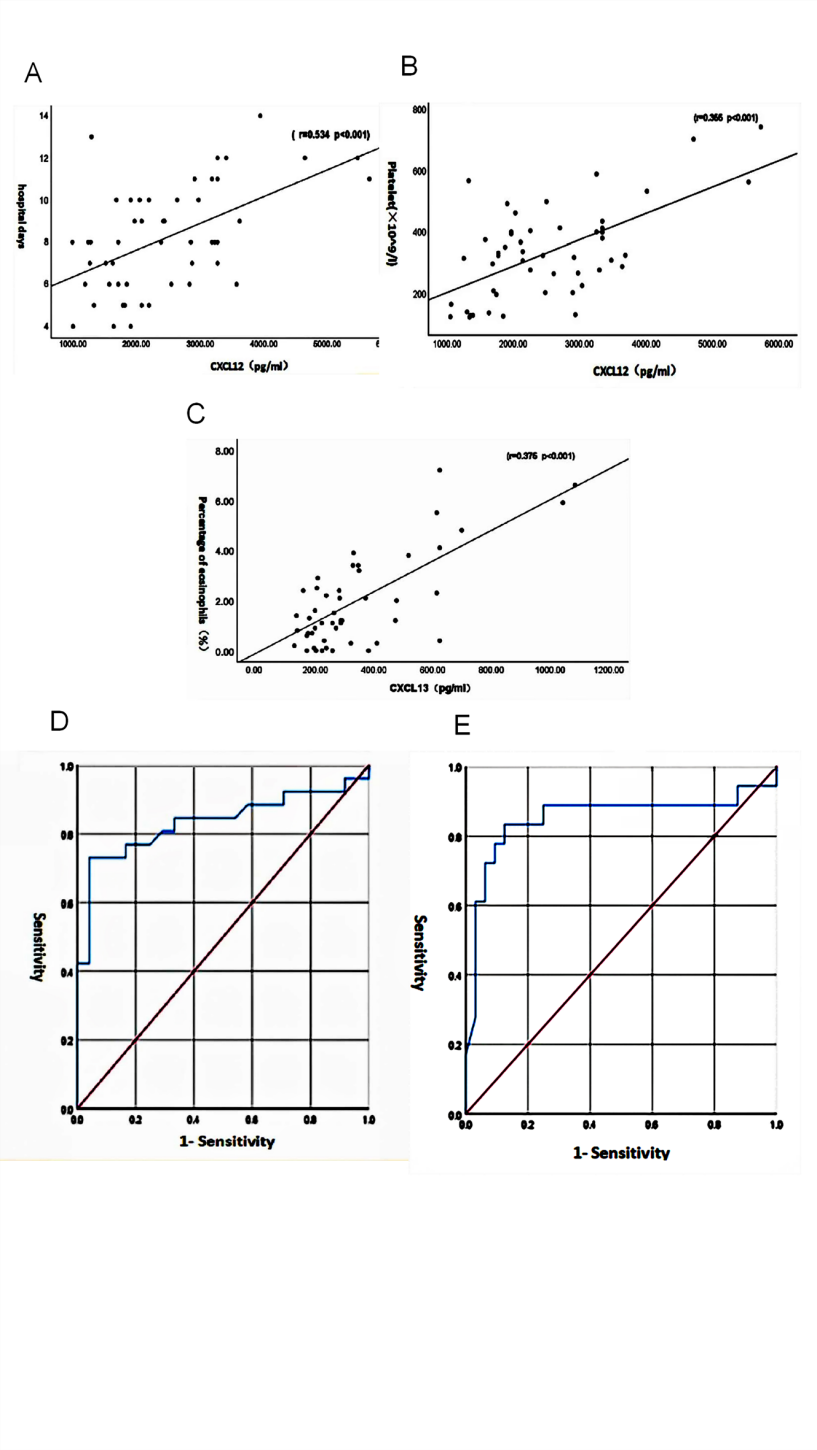
Correlation analysis of plasma CXCL12 and CXCL13 with clinical indicators and ROC curve analysis of association with disease conditions. (**A**–**C**) The plasma CXCL12 level was positively correlated with the length of hospital stay and platelet count, and the plasma CXCL13 level was correlated with the percentage of eosinophils. (**D**) ROC curve analysis of the relationship between CXCL12 and the severity of the disease. (**E**) ROC curve analysis of the relationship between CXCL13 and the occurrence of recurrent wheezing.

In children with RSV bronchiolitis, plasma CXCL12 levels showed a positive correlation with the length of hospital stay and platelet count (*r* = 0.534 and 0.355; *P* < 0.001). Additionally, plasma CXCL13 levels were positively correlated with the percentage of eosinophils (*r* = 0.375; *P* < 0.001). However, no significant correlations were observed between the plasma levels of CXCL2, CXCL12, CXCL13, CCL13, or CCL24 and other clinical indicators, including white blood cell count, C-reactive protein (CRP), Heparin-binding protein (HBP), Procalcitonin (PCT), Lactate dehydrogenase (LDH), lymphocyte subsets, total IgE, or pulmonary function parameters (Fig. 5A–C).

### Analysis of plasma chemokine risk factors and ROC curve for the severity of RSV bronchiolitis in children

Considering the severity of the disease as the dependent variable, and CXCL2, CXCL12, CXCL13, CCL13, and CCL24 as the independent variables, logistic regression analysis was performed. The results showed that CXCL12 was an independent risk factor for the severity of RSV bronchiolitis in children (*P* < 0.05) (Table 5). The ROC curve analysis showed that the area under the curve (AUC) value of the CXCL12 level for predicting the severity of the disease was 0.835 (95% CI 0.714–0.956), with a sensitivity and specificity of 73.1% and 95.8%, respectively. When at the optimal threshold of CXCL12, the predicted value was 2658.93 pg/ml (Fig. 5D).

**Table 5:** Binary Logistic regression analysis of risk of chemokines associated to severity of disease. **P < 0.01.

Chemokine	OR	95% CI	*P*
CXCL2	1.000	(0.999, 1.001)	0.407
CXCL12	1.002	(1.001, 1.004)	0.001**
CXCL13	0.997	(0.991, 1.002)	0.392
CCL13	1.003	(0.996, 1.011)	0.353
CCL24	1.002	(0.994, 1.009)	0.286

Considering the recurrence of wheezing as the dependent variable, and CXCL2, CXCL12, CXCL13, CCL13, and CCL24 as the independent variables, logistic regression analysis was carried out. The results showed that CXCL13 was an independent risk factor for the recurrence of wheezing in children with RSV bronchiolitis (95% CI 1.004–1.021, *P* < 0.05) (Table 6). The ROC curve analysis showed that the AUC value of the CXCL13 level for predicting the recurrence of wheezing was 0.851 (95% CI 0.711–0.991), with a sensitivity and specificity of 83.3% and 87.5%, respectively. When at the optimal threshold of CXCL13, the predicted value was 306.448 pg/ml (Fig. 5E).

**Table 6:** Binary logistic regression analysis of the risk of chemokines associated with recurrent wheeze. **P < 0.01.

Chemokine	OR	95% CI	*P*
CXCL2	1.000	(0.999, 1.000)	0.363
CXCL12	0.999	(0.998, 1.000)	0.207
CXCL13	1.013	(1.004, 1.021)	0.003**
CCL13	1.000	(0.999, 1.000)	0.363
CCL24	0.999	(0.998, 1.000)	0.207

## Discussion

RSV infection is the main cause of acute bronchitis in infants and young children. Some children may progress to severe illness or experience recurrent wheezing, which may even increase the risk of developing asthma later in life^12,13^. Therefore, elucidating the immunopathological mechanisms of severe disease and wheezing following RSV infection, and identifying biomarkers for early identification and prognostic assessment, hold significant clinical importance.

In recent years, the role of chemokines in the host response to RSV has garnered increasing attention. Studies have shown that chemokines involved in RSV infection exert chemotactic effects on inflammatory cells and may play a significant role in the pathophysiology of RSV bronchitis^14^. However, research on respiratory chemokines during RSV bronchitis remains limited^15^, warranting further exploration. In this study, through a screening process, we ultimately identified five chemokines—CXCL2, CXCL12, CXCL13, CCL13, and CCL24—that exhibited significant alterations in children with RSV bronchitis. Further correlation, multivariate, and ROC curve analyses revealed that aberrantly high expression of CXCL12 and CXCL13 is one of the core manifestations of RSV infection: elevated CXCL12 levels serve as an independent predictor for progression to moderate-to-severe RSV bronchitis, while CXCL13 is an independent predictor for the development of recurrent wheezing following RSV bronchitis.

CXCL12, also known as stromal cell-derived factor-1, exerts its biological functions primarily through the CXCL12/CXCR4 axis, which serves as the molecular basis of its activity^16^. This axis mediates the chemotactic recruitment of inflammatory cells to local tissues and regulates the release of inflammatory factors^17^, thereby playing a role in airway inflammatory diseases and airway remodeling processes^18^. This study confirms that CXCL12 levels are positively correlated with the duration of hospitalization and platelet counts, consistent with its role in regulating inflammatory factor release during respiratory viral infections^19,20^. Deeper mechanistic studies have revealed that during the late phase of RSV infection, high mobility group box 1 (HMGB1) forms a complex with CXCL12 and specifically recruits natural killer (NK) cells to the airways via the CXCR4 receptor, significantly exacerbating persistent airway inflammation and airway hyperresponsiveness (AHR). This provides direct molecular evidence for the development of wheezing symptoms in children with RSV infection^18^. Furthermore, Vanheule et al.^21^ demonstrated that the positively charged COOH-terminal region of CXCL12 exhibits high affinity for glycosaminoglycans (GAGs). Given that RSV is known to bind to GAGs, this suggests that CXCL12 may participate in the initial interaction between the virus and host cells^22^. Based on these findings, targeting the CXCL12/CXCR4 axis is considered a highly promising therapeutic strategy. For instance, in a murine model of RSV infection, the CXCR4 inhibitor AMD3100 significantly suppressed NK cell recruitment to the airways and alleviated airway inflammation and AHR^18^. Similarly, in models of allergic airway inflammation, AMD3100 mitigated pathological damage by downregulating molecules such as MMP-9 and ERK1/2^23^ or by inhibiting Th17/Tc17 immune responses^24^.

CXCL13, also known as B lymphocyte chemoattractant, primarily recruits B cells and also exerts migratory effects on a subset of T cells and macrophages, forming a signaling network that regulates the progression of various diseases^25^. Studies by Alturaiki et al. have confirmed that CXCL13 plays a significant role in the airway response to RSV infection, and its expression may be associated with cellular regions constituting lymphoid aggregates^26,27^. This study further revealed a positive correlation between the eosinophil percentage and the level of the pro-inflammatory chemokine CXCL13. We propose that RSV infection may activate the CXCL13/CXCR5 axis, leading to the recruitment of B cells to the site of infection and exacerbating mucosal damage through an enhanced humoral immune response. This effect aligns with the observed enrichment of the “B cell activation gene module” detected in transcriptomic analyses and plays a significant role in the imbalance of the airway immune microenvironment. Furthermore, CXCL13-mediated recruitment and activation of B cells may promote the deposition of local immune complexes in the airways, thereby increasing the risk of recurrent wheezing. Studies have confirmed that in an ovalbumin-induced model of allergic inflammation, anti-CXCL13 antibody treatment can attenuate inflammatory cell recruitment, formation of bronchus-associated lymphoid tissue (BALT), and airway inflammation^28^.

## Conclusions

CXCL12 and CXCL13 serve as auxiliary biomarkers for disease assessment and prognosis evaluation. A CXCL12 level exceeding 2658.93 pg/ml indicates progression to severe viral pneumonia, whereas a CXCL13 level above 306.448 pg/ml predicts recurrent wheezing in children with RSV bronchiolitis. Early monitoring of these chemokines facilitates treatment planning and wheezing risk prediction. However, as inflammatory factors, their diagnostic specificity is constrained by confounding conditions, individual variations, and lifestyle factors. Therefore, establishing clinical diagnoses requires combining these markers with other specific indicators.While, whether early intervention can be considered for children with RSV infection and elevated CXCL13 levels to reduce the probability of subsequent recurrent wheezing still requires further clinical exploration and verification.

## Data Availability

The raw data supporting the findings of this study are available in the OMIX repository under accession number OMIX009949. The raw sequencing data have been uploaded to [https://ngdc.cncb.ac.cn/account/active/zhanglinlin0610@126.com/20250424020341/b6b33ed06afee09cd652a1cb76604722/cd0e226fb820bc76e8fa261408024251] (Accession No.: OMIX009949: Chemokine Panel 2 Full Kit (RSV)). The anonymized cytokine detection data have been deposited as Supplementary Material MD5 e21072547f96cd66783016ffd98cc596 (Accession No.: OMIX009949) and made publicly available. Additional data generated and/or analysed during the current study are available from the corresponding author upon reasonable request. Please contact Dr. Zhengrong Chen at chen_zheng_rong@163.com for access.
